# Functional Overreaching During Preparation Training of Elite Tennis Professionals

**DOI:** 10.2478/v10078-011-0025-x

**Published:** 2011-07-04

**Authors:** Christian Thiel, Lutz Vogt, Meike Bürklein, Andreas Rosenhagen, Markus Hübscher, Winfried Banzer

**Affiliations:** 1 Goethe-University, Department of Sports Medicine, Frankfurt/Main, Germany

**Keywords:** racket sports, strength and conditioning mesocycle, overreaching markers

## Abstract

This case study evaluated the response of objective and subjective markers of overreaching to a highly demanding conditioning training mesocycle in elite tennis players to determine 1) whether players would become functionally or non-functionally overreached, and 2) to explore how coherently overreaching markers would respond.

Performance, laboratory and cardiac autonomous activity markers were evaluated in three experienced male tennis professionals competing at top 30, top 100 and top 1000 level before and after their strength and conditioning training was increased by 120, 160 and 180%, respectively, for 30 days. Every week, subjective ratings of stress and recovery were evaluated by means of a questionnaire.

After 74, 76 and 55 h of training, increases in V̇O_2_max (+8, +5 and +18%) and speed strength indices (+9, +23 and +5%) were observed in all players. Changes of maximal heart rate (−5, −6, +4 beats per minute), laboratory markers (e.g. insulin-like growth factor −26, −17, −9%; free testosterone to cortisol ratio −63, +2, −12%) and cardiac autonomous activity markers (heart rate variability −49, −64, −13%) were variable among the players.

Improved performance provides evidence that overreaching was functional in all players. However, several overreaching markers were altered and these alterations were more pronounced in the two top 100 players. The response of overreaching indicators was not coherent.

## Introduction

Professional tennis players compete in about 50 to 90 competitive matches per year in the Association of Tennis Professionals (ATP) tour. Accordingly, volume and intensity of conditioning need to be reduced during the season from January to mid-November, and a relatively short period of 5–6 weeks at the end of the year offers the best opportunity to substantially improve motor abilities and get into shape for the up-coming season. Players follow a demanding conditioning regime and undergo training stimuli which may differ in volume and intensity from the physiological demands imposed by the matches played throughout the season.

Functional overreaching is defined as a short-term decrement in performance as a result of increased training stress. It is a usual part of the training process of elite athletes and its recovery to regular performance occurs within a few days (Meeusen et al., 2006). Non-functional overreaching is also transient, but may take several days or a few weeks to restore. Still, it is often considered a normal outcome for elite athletes (Halson & Jeukendrup, 2004). Overtraining is a persisting performance decrement which takes several weeks or months to restore and may seriously harm the athlete’s health (Halson & Jeukendrup, 2004; Meeusen et al., 2006). While alterations of several performance-related, endocrine, immunological, vegetative and neuropsychological indicators as well as of physiological exercise responses have been associated with overreaching and over-training (Urhausen & Kindermann, 2002), there is no evidence that diagnostic tools can discern acute functional from non-functional overreaching and overtraining (Halson & Jeukendrup, 2004; Meeusen et al., 2006).

This lack of widely accepted markers may be one among other reasons why the prevalence of overreaching in professional tennis is currently unknown. No study has yet attempted to monitor overreaching in high-level tennis players. Notwithstanding, sports medicine physicians have called for a rigorous assessment of overreaching indicators in tennis (König et al., 2001; Kibler et al., 1992). However, unlike in team sports and various individual sports, professional tennis players rarely practice in large groups at the same location for a prolonged time. This makes large and elaborate longitudinal evaluations time-consuming and costly. Therefore, it seems reasonable to start with exploring the association between potential overreaching markers and performance changes in a small sample of elite players at different levels.

Thus, the current study evaluated the impact of an intense conditioning training mesocycle on common overreaching indicators in three elite tennis players competing at different levels. The following research questions were formed:

1. Is there any evidence that the athletes monitored were non-functionally overreached?
2. Is the response of potential overreaching markers coherent, or does this response differ in magnitude or direction between the indicators employed?

## Methods

Three healthy male professional tennis players (26–28 years, BMI 21.8–24.8 kg/m^2^, 7–10 years of ATP tour experience, hard-court specialists) had signed written informed consent to participate in this study which was performed in accordance with the ethical standards reported by Harris & Atkinson (Harriss & Atkinson, 2009) and meets the standards as described in the Declaration of Helsinki. In a longitudinal design, performance and indicators which have been found to be related to overreaching (Halson & Jeukendrup, 2004; Urhausen & Kindermann, 2002) were monitored before (mid November) and after the preparation period (late December). Measurements were taken at the same time (morning) and sequence (heart rate variability, blood sampling, speed strength and cardiorespiratory fitness) and under similar conditions, after 24h of abstinence from exercise, caffeine and alcohol and an overnight fast. Additionally, mood, perceived stress and recovery (psychophysical state) were evaluated weekly by questionnaire.

### Cardiac autonomous control

Heart rate variability (HRV), indicative of cardiovascular autonomic balance, has been reported to be related to overreaching (Mourot et al., 2004; Uusitalo et al., 2000). After 10 minutes of rest, HRV was assessed for 5 minutes while players were lying in the supine position in a quiet, temperature-controlled room (22–24°C), using the VarCor PF5© system (Pantalus GmbH, Rheinmünster, Germany). Recording, filtering and analysis of heart rate followed standard procedures as previously published by Bürklein et al (Bürklein et al., 2005). All procedures including raw data processing followed recommendations of the Task Force of the European Society of Cardiology and the North American Society of Pacing and Electrophysiology (Task Force of the European Society of Cardiology and the North American Society of Pacing and Electrophysiology, 1996).

### Blood sampling and biochemical analyses

Blood samples were collected from the ante-cubital vein at rest between 8.00 and 9.00 a.m. Samples were stored at 2–8 °C and analyzed by automated clinical systems within 2.5 hours. Plasma urea was measured using an enzymatic calorimetric endpoint method (Hitachi/Roche 917, Mannheim, Germany). Serum total testosterone and cortisol were assessed by competitive immunoassay using direct chemiluminescence (Advia Centaur, Siemens, Erlangen, Germany). Determinations of IGF-I were made with a solid phase, enzyme-labelled chemiluminescent immunometric assay (Immulite 2000, Siemens, Erlangen, Germany).

### Cardiorespiratory fitness test

Athletes performed an incremental exercise test on a motor driven treadmill (Quasar med, HP Cosmos, Nussdorf, Germany). Protocol, measurements and data analysis have been described in detail elsewhere (Banzer et al., 2008). Shortly, at a gradient of 0%, the initial speed of 8.5 km/h was increased by 1.5 km/h every four minutes until voluntary exhaustion. Lactate concentration in capillary blood from ear-lobe was determined immediately after each stage by photometric analysis (Arcray Lactate Pro LT-1710, Kyoto, Japan). Pulmonary gas exchange was measured breath-by-breath using a mobile indirect calorimetry system (Oxycon Mobile, Viasys Healthcare, Würzburg, Germany) after sufficient warm-up and standardized calibration.

### Speed-strength assessment

The ability of the neuromuscular system to overcome resistance with the highest contraction speed possible (explosive strength) and to produce the greatest impulse in the shortest time (stretch-shortening cycle strength) was assessed by countermovement jump (CMJ) and by the drop jump (DJ), respectively, following the protocol suggested by Logan et al. (Logan et al., 2000). Single-leg CMJs were performed on a timing mat with hands on hips and the free leg fixed. Jump height (mean of dominant & non-dominant leg) was calculated based on flight time. The two-leg DJs were performed from 32 cm height with hands on hips. Jump height [cm] was divided by contact time [s] to compute the DJ performance index (Hennessy & Kilty, 2001). Both for CMJ and DJ, the best of 5 attempts was used to represent speed strength ability.

### Psychophysical state

The German version of the EBF-52 SPORT questionnaire (Erholungs-Belastungs-Fragebogen – Recovery-Stress Questionnaire, RESTQ) (Kellmann & Kallus, 2001) was used to measure the psychophysical state. The EBF-52 consists of 52 items organized in 4 subcategories relating to general stress (e.g. “I had a bad temper”), general recovery (“I have slept soundly”), sports-specific stress (“I felt burned out by my sports”) and sports-specific recovery (“I was physically relaxed”). Athletes are asked how frequently they have experienced a particular mood or attitude within the last three days. Each item scores between 0 (never) and 6 (always) and mean scores are computed for each of the four subcategories. A regular profile is characterized by stress scores below 2 and a good recovery is considered to be indicated by recovery scores of 4 or higher (Kellmann & Kallus, 2001). Reliability of the EBF-52 is r =.79 (Kellmann, 2000). Single EBF-52 items correlate with the Profile of Mood Scores (POMS) global score (r =.24-.81) (Kellmann & Kallus, 2001).

### Training programme

The conditioning mesocycle was designed individually according to the specific aims and initial test results, and left room for weekly adjustments. The primary aim of the top 30 player was to improve endurance and speed endurance, while the top 100 player was slightly more focused on developing strength and speed strength. The top 1000 player, whose performance had been hampered by a chronic foot injury in the preceding months, aimed at regaining his previous fitness level. The conditioning training included endurance, speed and agility, as well as strength and sensorimotor training.

### Endurance

- The intensity of endurance training was prescribed relative to the individual lactate anaerobic threshold (AnT) as determined during the initial cardiorespiratory fitness test on the treadmill.
  ○ Steady-state moderate aerobic training: 70–85% of AnT velocity
  ○ Vigorous steady-state exercise and moderate interval training: 85–100% of AnT velocity
  ○ High-intensity intervals: Between AnT and V̇O_2_max velocity

### Power and agility

- Short sprints, plyometric training, agility drills and medicine ball throws with complete recovery in between sets (alactic)
- Cross training (soccer, karate) and speed endurance sprints characterized by an incomplete recovery between sets (lactic)

### Strength and sensorimotor training

- Sensorimotor training for legs, trunk and shoulders (primarily balance exercises); moderate resistance exercises for shoulder and trunk
- Whole-body strength training
  ○ Strength endurance: 3–5 × 15–20 repetitions, resistance 40–55% of 1RM
  ○ Hypertrophy: 2–4 × 10–12 repetitions, 70–80% of 1RM
  ○ Neuromuscular adaptation: pyramid system, 3–6 × 3–8 reps 80–95% of 1RM

For regeneration, athletes underwent massage by a physiotherapist (1.5–4.0 h/wk), stretched (0.5–1.5 h/wk), performed low-intensity ergometer cycling (0.5–1.0 h/wk) and stayed in the Sauna 1–2 times/wk.

The volume of conditioning was decreased during the last two weeks, and especially during the last week of the preparation period, while on-court tennis practice time was increased. Volume and intensity of training load were reduced on Thursdays, and Sunday was off. If possible, the three athletes practiced together as a group. All conditioning training sessions were guided, monitored and documented by an experienced conditioning coach.

Overall, conditioning training volume in the preparation period was increased by 120, 140 and 180% (top 30, top 100, top 1000 player) as compared to the training volume the players had completed on their own during the competitive season in the preceding months.

### Data analysis

Because this case study involved three subjects only, we solely focused on descriptive statistics. Data are presented either individually for each player, or as a range.

## Results

**Figure 1:**
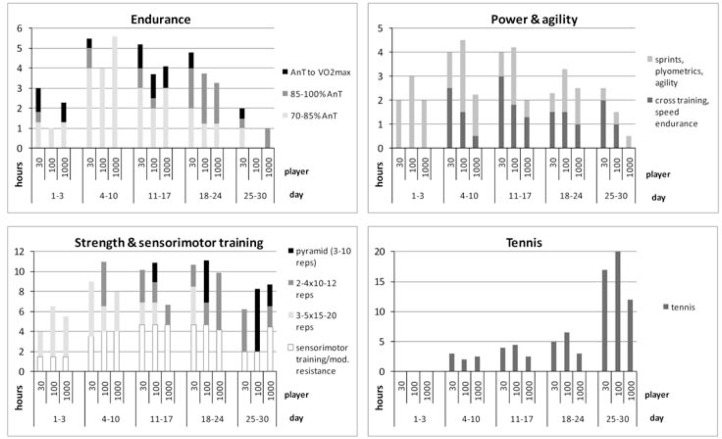
Individual training load in tennis players over 30 days of preparation training. 70–85% AnT: Steady-state moderate aerobic training at 70–85% of the velocity at the individual anaerobic threshold; 85–100% AnT: Vigorous steady-state exercise and moderate interval training; AnT to V̇O_2_max: High-intensity intervals; reps: repetitions; mod. resistance: moderate resistance exercises for shoulder and trunk

Athletes completed 74, 76 and 55 h of conditioning, corresponding to 95, 96 and 93% of their scheduled training sessions. The amount and quality of endurance, strength, sensorimotor, speed and agility, as well as on-court tennis training can be depicted from figure 1.

After 30 days of training, body weight and percent body fat remained unchanged in the top 30 (baseline: 74.5 kg and 9.5%, respectively) and the top 100 player (72.3 kg, 7.3%), while the top 1000 player (85.3 kg, 19.4%) lost 1.5 kg of body weight and reduced body fat by 1.7%. Aerobic capacity and speed strength were improved in all players. In the month following the preparation period, the top 30 player improved his ATP entry ranking by 15 positions, whereas the ranking of the top 100 (−4) and top 1000 player (−15) decreased.

**Figure 2:**
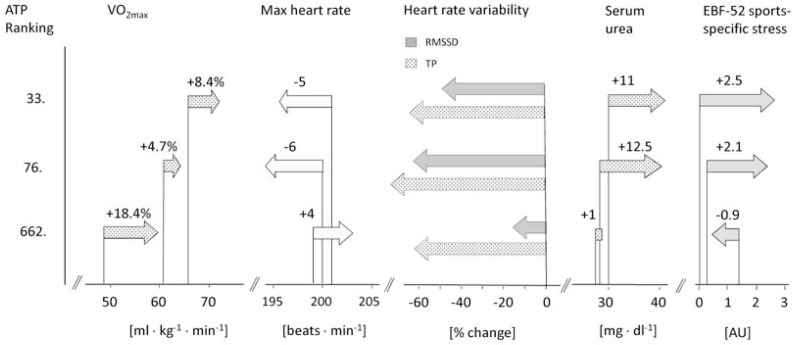
Changes of performance and of overreaching markers in differently ranked tennis players after intense 30-day preparation. ATP: Association of Tennis Professionals; V̇O_2_max: maximum oxygen consumption; RMSSD: root mean square of successive differences; TP: total power

Resting heart rate was increased and heart rate variability was reduced after the training period in all players. Lactate and heart rate responses to maximum exercise stress as well as alterations of hormonal parameters were variable among the three players (table 1, figure 2).

**Figure 3:**
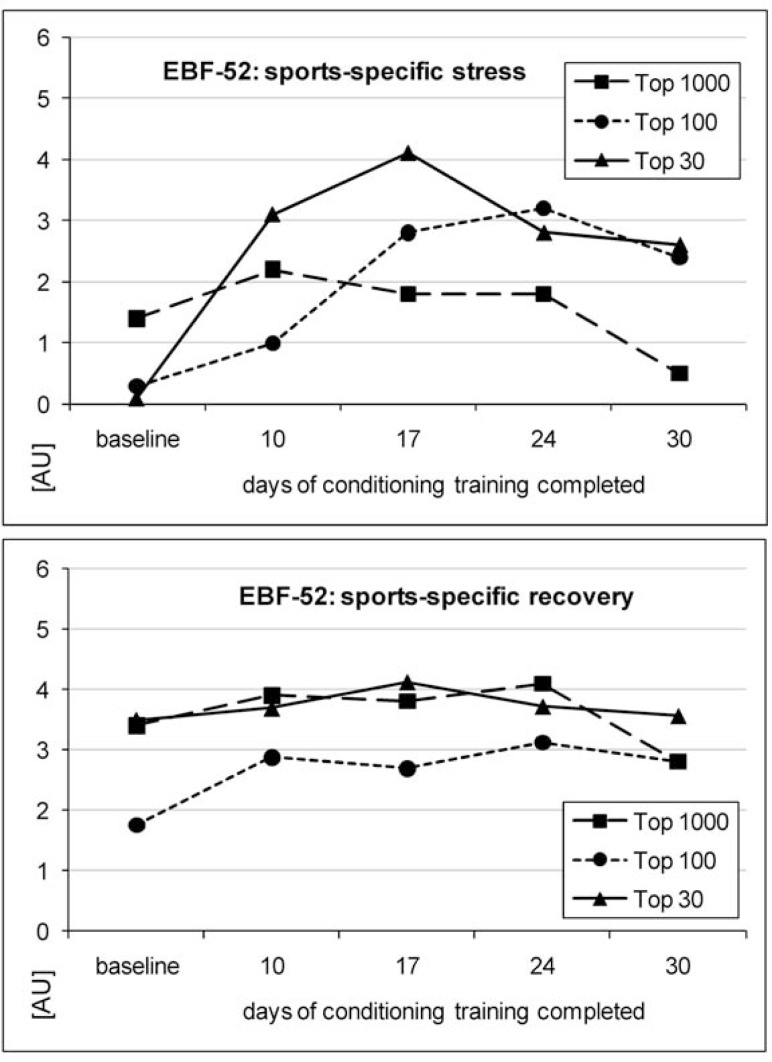
Sports-specific stress and sports-specific recovery over a 30-day preparation period in elite tennis players.

In terms of psychophysical state, the EBF-52 scores of the general stress subcategory remained low in all players (0.4–1.7), while general recovery was constantly rated moderate (3.0–4.0; individual data of players not shown). In the top 30 and the top 100 players, perceived sports-specific stress increased to levels considered to indicate over-reaching (4.1 and 3.2, respectively) but remained low in the top 1000 player (2.2). Sports-specific recovery showed small variation throughout the preparation period and was constantly rated between 1.8 and 4.1 (figure 3).

## Discussion

Non-functional overreaching is a transient decrement in performance capacity despite increased training stress which restores within weeks (Halson & Jeukendrup, 2004; Meeusen et al., 2006). For the first time, the current case study assessed the impact of an intense tennis conditioning training on common overreaching indicators in elite tennis professionals. The present findings do not provide evidence that the tennis professionals were overreached because all performance markers had improved or were unchanged. However, changes in many, but not all overreaching markers over the course of the 30 days of intense preparation conditioning indicate a high training stress typical of functional overreaching.

The higher performance capacity of the top 100 and especially the top 30 player allowed them to conduct higher training volumes (+25%) and higher absolute intensities than the top 1000 player. These higher workloads seem to have resulted in stronger deflections of several overreaching markers, even though performance was not compromised.

Interestingly, the top 1000 player increased his V̇O_2_max by an extraordinary 18% within 30 days. Compared to his personal best V̇O_2_max recorded mid November in previous years (59 ml·kg^−1^·min^−1^), the player had entered the conditioning programme in relatively poor shape due to his foot injury (49 ml·kg^−1^·min^−1^). It is well known that after prolonged training interruption, aerobic fitness may be regained more rapidly. However, subject effort may also have been greater in the second test than in the first, even though criteria for attaining V̇O_2_max were fulfilled.

Regarding the immediate response to maximum exercise, the two higher ranked players showed reduced maximal heart rates (HR max) despite enhanced performance. A reduced HR max has been found in highly strained or over-reached athletes (Jeukendrup et al., 1992; Lehmann et al., 1991; Hedelin et al., 2000; Urhausen et al., 1998a), as confirmed by a recent review (Bosquet et al., 2008). Only the top 30 player reached a lower maximal blood lactate concentration compared to the first test, which has also been observed in fatigued or overreached athletes (Callister et al., 1990; Urhausen et al., 1998a; Hedelin et al., 2000; Costill et al., 1988; Jeukendrup et al., 1992). Athletes had one day of rest before the test, but it is still possible that the high volume of training may have induced a slight glycogen depletion, which would explain reduced maximal lactate concentration. In this light, observing maximal blood lactate concentration does not seem particularly useful for overreaching monitoring.

Sports-specific perceived stress increased markedly in the first half of the preparation period in both top 100 players before displaying a plateau (top 100 player) and slightly decreasing (top 30 player), whereas it did not change substantially in the first weeks and decreased in the last weeks in the top 1000 player. In other studies, overreached athletes have shown a deterioration in mood state, psychological distress and sleep disorders before a drop in performance (Halson et al., 2002; Jeukendrup et al., 1992; Fry et al., 1992; Urhausen et al., 1998a). However, these findings have also been observed before improved performance (O'Connor et al., 1996; Morgan et al., 1988). In agreement with the latter studies, our results suggest that while questionnaires related to psychophysical state and mood, or more specifically, the EBF-52 may be an important tool for athletes to provide feedback and for coaches to monitor training load, they cannot discern between functional and non-functional overreaching in cross-sectional analyses. However, they may be valuable for the individual prediction of an impending non-functional overreaching, particularly because they can easily be administered on a regular basis.

Serum urea remained within values considered normal (10–50 mg·dL^−1^) in all players (Gardner & Scott, 1980; Hartmann & Mester, 2000) despite an increase in the two top 100 players. However, due to large interindividual variation, setting a fixed numerical serum urea value indicative of overreaching may be problematic (Hartmann & Mester, 2000). An increase in serum urea has been reported to mark enhanced protein catabolism and stimulated gluconeogenesis linked to heavy training and functional overreaching (Kreider et al., 1998; Urhausen et al., 1995; Lemon & Mullin, 1980), while findings have been inconsistent in relation to non-functional overreaching (Halson et al., 2002). Testosterone-cortisol ratio (FTCR), correlated with maximal strength performance and suggested as a marker of anabolic-catabolic balance (Adlercreutz et al., 1986), was reduced by 63% in the top 30 player but remained largely unchanged in the other players. Testosterone and cortisol have opposing effects on muscle metabolism and protein synthesis. Testosterone, an anabolic hormone, plays a significant role in the growth and maintenance of skeletal muscle, bone, and red blood cells. Cortisol, a catabolic hormone secreted in response to physical and psychological stress, facilitates the conversion of amino acids into glucose and glycogen (Kreider et al., 1998). An FTCR of 3.5%, or a 30% drop in FTCR have been proposed to indicate overreaching (Adlercreutz et al., 1986). Both conditions were met by the top 30 player even though he was not overreached. Other authors have reported similar findings (Lehmann et al., 1992; Urhausen et al., 1998a; Urhausen et al., 1998b; Mackinnon et al., 1997; Uusitalo et al., 1998; Jeukendrup et al., 1992). Insulin-like growth factor-I (IGF-I), a growth hormone associated with overreaching which induces protein synthesis and blocks muscle atrophy, was reduced in all three players, but remained within values considered normal (125-460 μg·l^−1^) (Koistinen et al., 1996; Rietjens et al., 2005; Kreider et al., 1998).

An elevated resting heart rate, observed in all three tennis professionals, has been reported in highly strained athletes (Dressendorfer et al., 2000; Stone et al., 1991) and suggested to be indicative of functional overreaching (Bosquet et al., 2008), but prospective studies have not been able to link a higher resting heart rate with nonfunctional overreaching (Achten & Jeukendrup, 2003; Fry et al., 1992; Lehmann et al., 1992; Urhausen et al., 1998a; Bosquet et al., 2008). Shifts in heart rate variability (HRV), indicative of cardiovascular autonomic balance, are considered a sign of fatigue after intense prolonged practice or competition (Bosquet et al., 2008; Earnest et al., 2004; Iellamo et al., 2002). HRV time and frequency-domain parameters were both reduced to a large extent in all three ATP players. In athletes reported as overreached, HRV was reported reduced (Mourot et al., 2004), inconsistently changed (Uusitalo et al., 2000), or unchanged (Hedelin et al., 2000). However, not all of these studies assessed performance so that it is unclear whether athletes were indeed overreached.

Limitations in the current case study obviously comprise the small sample size and the lack of a longitudinal tracking of the markers. Also, tennis performance is conditioned by many diverse factors such as physiological, anthropometric and biomechanical characteristics, technical skills, motivation, and the occurrence of acute or chronic injuries, so that a comparison between the three different tennis players is difficult, as is the extrapolation of the presented results.

Even so, the variability observed in the response of various overreaching markers seems indicative of the difficulties related to the diagnosis of overreaching states. Attempts to identify reliable, specific and sensitive parameters for nonfunctional overreaching and overtraining have not been successful so far (Halson & Jeukendrup, 2004; Urhausen & Kindermann, 2002). The challenge remains to determine valid thresholds which can be used in daily training routine to individually adapt training intensity and regeneration cycles, and which help to prevent an unwanted transition from functional to nonfunctional overreaching resulting in prolonged performance deterioration. In our study, psychophysical state (EBF-52), heart rate variability and serum urea showed a coherent reaction. Congruent with published results (Urhausen & Kindermann, 2002), these indicators changed parallel to the increase in training load, well before a drop in performance occurred. Thus, such indicators do not qualify as a marker of non-functional over-reaching, but they might be used as predictors of non-functional overreaching. Longitudinal tracking studies using short measurement intervals should determine whether changes in these indicators allow discrimination of a later onset of an overreaching. These investigations should also incorporate promising new early-warning signs like psychomotor speed (Rietjens et al., 2005; Nederhof et al., 2006).

To conclude, enhanced performance shows that functional overreaching occurred in three professional tennis players after a prolonged intense conditioning training mesocycle. The tennis players tolerated up to 75 hours of individualized training within 30 days. Endocrine, vegetative and psychophysical markers as well as the physiological response to exercise did not show a coherent response, but the alterations were generally more pronounced in the Top 100 players. Using single markers for the identification of impending nonfunctional overreaching in sports practice may lead to false alarms. To identify athletes who are failing to cope with the stress of training, coaches and medical staff might need to monitor performance, physiological, biochemical, vegetative and psychological markers on a regular basis.

## Figures and Tables

**Table 1:** Performance and markers of overreaching before and after 30-day preparation training in elite tennis players.

		**ATP ranking**	Top 30		Top 100		Top 1000	
			pre	post	pre	post	pre	post
Performance	V̇O_2max_	[ml·kg^−1^·min^−1^]	66	72	61	64	49	60
	DJ index	[cm/s]	183	190	175	229	165	193
	single-legged CMJ	[cm]	20.2	22.0	19.0	23.3	12.1	12.8

Physiological response at max. exertion	Max. Lactate	[mmol·l^−1^]	16.1	9.9	9.7	12.7	8.4	11.2
	Max. HR	[beats·min^−1^]	201	196	200	194	199	203

Hormonal parameters	IGF-I	[μg·l^−1^]	183	135	177	147	235	215
	FTCR	[%]	9.2	3.4	4.0	4.1	4.3	3.8
	Serum urea	[mg·dl^−1^]	30.2	41.0	27.5	40.0	26.0	27.0

Heart rate variability	Resting HR	[beats·min^−1^]	52	57	50	54	47	53
	RMSSD	[ms]	77	39	77	28	65	56
	TP	[ms^2^]	1755	610	2185	605	2308	870

ATP, Association of Tennis Professionals; VO_2max_, maximum oxygen consumption; DJ, drop jump; CMJ, counter movement jump; HR, heart rate; IGF-I, insulin-like growth factor-I; FTCR, free testosterone to cortisol ratio; RMSSD, root mean square of successive differences; TP, total power.
